# Correction: Taniuchi et al. The Combination of Binding Avidity of Ovomucoid-Specific IgE Antibody and Specific IgG4 Antibody Can Predict Positive Outcomes of Oral Food Challenges during Stepwise Slow Oral Immunotherapy in Children with Hen’s Egg Allergy. *Nutrients* 2023, *15*, 2770

**DOI:** 10.3390/nu17040635

**Published:** 2025-02-11

**Authors:** Shoichiro Taniuchi, Rika Sakai, Takahiro Nishida, Meguru Goma, Masatoshi Mitomori, Aya Imaide, Masahiro Enomoto, Masamitsu Nishino, Yo Okizuka, Hiroshi Kido

**Affiliations:** 1 Department of Pediatrics, Takatsuki General Hospital, Osaka 569-1192, Japan; 2 Division of Enzyme Chemistry, Institute for Enzyme Research, Tokushima University, Tokushima 770-8501, Japan

The authors wish to make the following correction to this paper [1].

**Error in Figure Legend**

Figure 3. (**c**) binding avidity of OVM-sIgE (1/IC_50_) (nM) is incorrect and has been corrected to (**c**) OVM-sIgE (IC50) (nM).

Figure 4. avidity of OVM-sIgE (1/IC_50_) (nM) is incorrect and has been corrected to avidity of OVM-sIgE (IC_50_) (nM).

**Error in Figure 3** **c**

Figure 3c title: 1/IC_50_ is incorrect and has been corrected to IC_50_.

Labels for vertical axis: I/IC_50_ (nM) is incorrect and has been corrected to IC_50_ (nM).

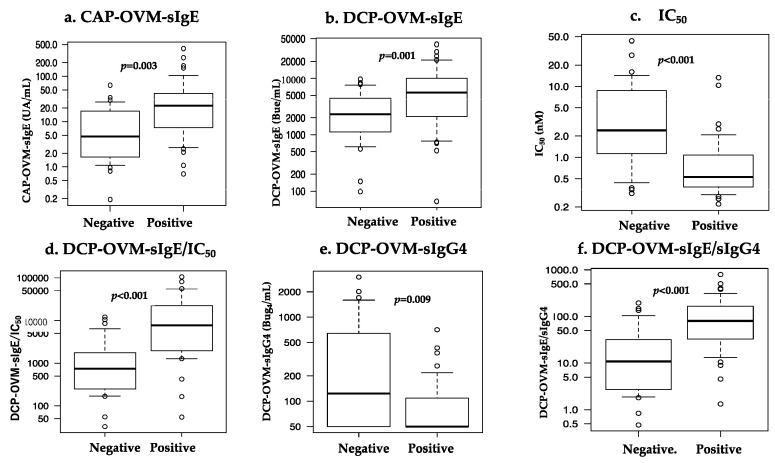

**Error in Figure 4**

The content in the figure: 1/IC_50_ is incorrect and has been corrected to IC_50_. I/IC_50_ (nM) is incorrect and has been corrected to IC_50_ (nM).

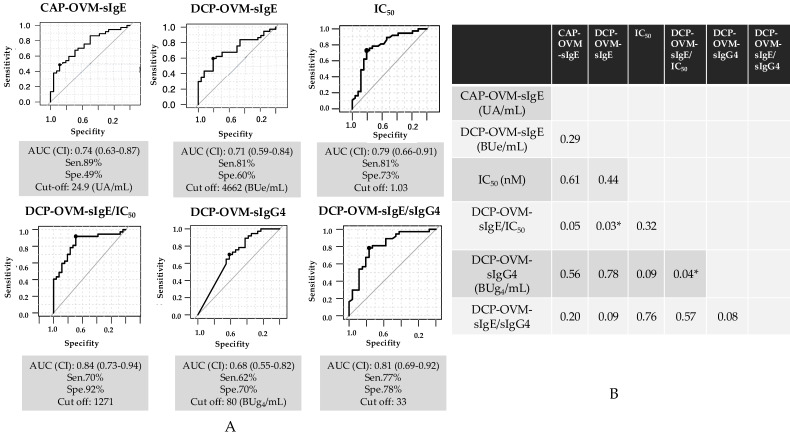

**Error in Table 3**

Line 4: I/IC_50_ (nM) is incorrect and has been corrected to IC_50_ (nM).

**Error in Table 4**

Line 4: 1/IC_50_ (nM) is incorrect and has been corrected to IC_50_ (nM).

**Error in Abstract**

In the 5th sentence: 1/IC_50_ is incorrect and has been corrected to IC_50_.

The authors apologize for any inconvenience caused and state that the scientific conclusions remain unaffected. The original publication has also been updated.
